# Asymmetric Jetting during the Impact of Liquid Drops on Superhydrophobic Concave Surfaces

**DOI:** 10.3390/mi13091521

**Published:** 2022-09-14

**Authors:** Chengmin Chen, Hongjun Zhong, Zhe Liu, Jianchun Wang, Jianmei Wang, Guangxia Liu, Yan Li, Pingan Zhu

**Affiliations:** 1 Energy Institute, Qilu University of Technology (Shandong Academy of Sciences), Jinan 250100, China; 2 School of Energy and Power Engineering, Qilu University of Technology (Shandong Academy of Sciences), Jinan 250100, China; 3 Jinan Key Laboratory of High-Performance Industrial Software, Jinan Institute of Supercomputing Technology, Jinan 250100, China; 4 Department of Mechanical Engineering, City University of Hong Kong, Hong Kong 999077, China

**Keywords:** droplet impact, superhydrophobic surface, asymmetric jetting, droplet manipulation

## Abstract

The impact of liquid drops on superhydrophobic solid surfaces is ubiquitous and of practical importance in many industrial processes. Here, we study the impingement of droplets on superhydrophobic surfaces with a macroscopic dimple structure, during which the droplet exhibits asymmetric jetting. Systematic experimental investigations and numerical simulations provide insight into the dynamics and underlying mechanisms of the observed phenomenon. The observation is a result of the interaction between the spreading droplet and the dimple. An upward internal flow is induced by the dimple, which is then superimposed on the horizontal flow inside the spreading droplet. As such, an inclined jet is issued asymmetrically into the air. This work would be conducive to the development of an open-space microfluidic platform for droplet manipulation and generation.

## 1. Introduction

Understanding the dynamics of impacting droplets on nonwetting surfaces is of both scientific and technological importance, such as spraying crops with pesticides [1], spray cooling of hot surfaces [2], 3D inkjet printing of micro/nanostructures [3], shedding virus-laden aqueous droplets away for the anti-pathogen purpose [4], and manipulating droplets with open-space microfluidics [5]. Droplet impact is ubiquitous on solid surfaces with various features, including inclined surface [6], rough surface [7], micro-channels [8], curved surface [9], micro-cellular surface [10,11], heated surface [12], cold surface [13,14], and nanoparticles-coated surfaces [15], to name a few. Varying the surface properties can significantly alter the behaviors and dynamics of droplet impact, such as splashing, spreading, bouncing, jetting, and bubble encapsulation [16,17,18,19,20].

The jetting phenomenon has been previously identified when liquid droplets impact both hydrophilic [21,22] and hydrophobic [23] surfaces. Previous studies showed that jetting is induced by the collapse of the air cavity formed by the deformation of the drop at impact [24,25,26]. Detailed studies on the mechanism of jetting formation can be found in several recent works [27,28,29]. Apart from flat hydrophobic surfaces, jetting also occurs on superhydrophobic surfaces with tailored structures, such as ice-leaf-inspired grooved superhydrophobic surfaces [30], superhydrophobic surfaces with anisotropic surface patterning [31], superhydrophobic copper meshes [32], oblique surfaces [24] and artificial dual-scaled superhydrophobic surfaces [33]. The previously reported jetting is symmetric, which is vertical to solid surfaces. However, asymmetric jetting that is inclined to solid surfaces is yet to be observed.

Here, we report asymmetric jetting when water droplets eccentrically impact a macro-sized dimple on superhydrophobic surfaces. The jetting velocity depends on the Weber number (We) and the impact position of the droplet, as demonstrated by experimental results. In parallel, numerical simulations reveal the internal flow field, pressure field, and momentum variation of the droplet. The combination of experimental and numerical studies shed light on the dynamics and mechanism of asymmetric jetting during droplet impact.

## 2. Experimental Methods

**Figure 1:**
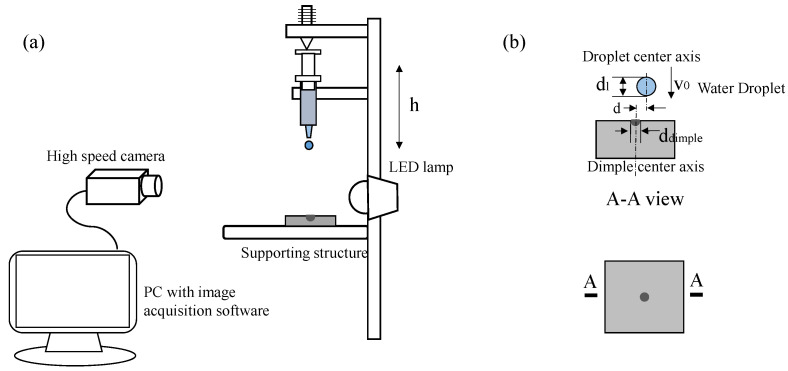
(**a**) Schematic of the experimental setup for droplet impact, (**b**) details of the solid surface.

The experimental setup is illustrated in Figure 1. Water droplets were generated from blunt syringe tips. The droplet diameter (*d_l_*, as shown in Figure 1b), which depends on the size of the syringe tips, was about 2.40 ± 0.05 mm. Following its detachment from the syringe tip, the droplet was accelerated by gravity and then impacted the superhydrophobic surface. The impact velocity (*v*_0_) of the droplet was changed by adjusting the height of the syringe tips, as varied from 0.5 m/s to 0.8 m/s. A copper surface was used in the experiments, on which a hemispherical dimple with a diameter of *d*_dimple_ = 1.2 mm was excavated. The eccentric distance (*d*, as shown in Figure 1b) stands for the horizontal distance between the center of the droplet and the center of the dimple. The relative eccentric distance (*e*) was defined as *e* = *d*/*d*_dimple_. The surface was cleaned with acetone and ethanol and then coated with candle soot for superhydrophobicity. The candle soot was deposited by exposing the surface to the outer flame of a burning candle for 3–5 s to ensure that the surface areas were fully covered by the candle soot particles. The water contact angle was measured by an Optical Surface Analyzer OSA200 (Ningbo NB Scientific Instruments Co., Ltd., Ningbo, China). The apparent contact angle was about 145.3° (Figure S1 in Supplemental Materials), averaged from three measurements. The sliding angle was 8° and the contact angle hysteresis (the difference between the advancing angle and the receding angle) was about 2.7°.

A high-speed camera (Miro M310, Phantom) was used to record the impact process at 4000 frames per second. The camera was placed parallel to the horizontal surface to obtain the side-view images and videos of impacting droplets. An LED lamp with a diffuser was used for illumination. The recorded images and videos were analyzed by the Phantom camera control software obtained from the camera supplier.

In general, the impact dynamics were characterized by the Weber number defined as the ratio of inertial to surface tension forces (Equation (1)), the Reynolds number defined as the ratio of inertial to viscous forces (Equation (2)), the Bond number defined as the ratio of gravitational to surface tension forces (Equation (3)) and the capillary number defined as the ratio of viscous to surface tension forces (Equation (4)) [34,35,36,37]. Here, ρ, $v_{0}$, $d_{l}$, σ, and μ are the density, the impact velocity, initial droplet diameter, the surface tension and the viscosity of the liquid, respectively. Water droplets were used in experiments, of which the density is 1000 kg/m^3^, the surface tension is 0.072 N/m and the viscosity is 0.001003 Pa·s.

(1) $$\mathrm{We}=\rho v_{0}{}^{2}d_{l}/\sigma$$

(2) Re=ρdlv0/μ

(3) $$\mathrm{Bo}=\rho gd_{l}^{2}/4\sigma$$

(4) $$\mathrm{Ca}=\mu v_{0}/\sigma$$

## 3. Asymmetric Jetting Phenomenon

**Figure 2:**
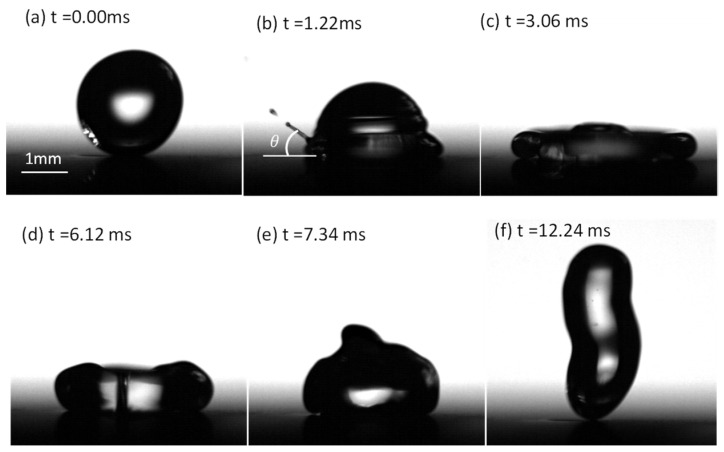
Snapshots showing a drop impacting the dimpled surface at We = 23.41. (**a**) Start of droplet impact at *t* = 0.00 ms. (**b**) Satellite droplets issued at the end of the Jetting. (**c**) Droplet spreading to its maximum diameter. (**d**,**e**) Asymmetric morphology of the droplet during retraction. (**f**) The rebound of the droplet from the surface.

The behavior of a droplet impacting the dimpled surface showed a remarkable difference from previous observations of droplet impact on flat superhydrophobic surfaces. Figure 2 presents snapshots of a droplet eccentrically impinging the dimpled surface, where the eccentric distance *d* = 0.5$d_{l}$, We = 23.41, Re = 1974.10, Bo = 0.20 and Ca = 0.01. The high values of We and Re indicated that inertial forces dominated over capillary and viscous forces during droplet impact, while the low values of Bo and Ca implied that the influences of gravitational and viscous forces were negligible compared with surface tension forces in this study. In Figure 2a, at *t* = 0 ms, the droplet contacted the dimpled surface, with We = 18.97. After contact, the droplet first spread when the time was less than 1.22 ms (Figure 2b) and it fully covered the dimple at *t* ~ 1.22 ms when an inclined jet was issued from the side of the droplet. The jetting angle (θ in Figure 2b and Figure S2 in Supplemental Materials) was about 45° relative to the horizontal plane of the surface. Afterwards, the droplet adopted asymmetric morphology during the spreading and contracting processes. As a result, the bouncing direction of the droplet could be well-controlled by changing the impact position around the dimple, as we identified previously [38].

**Figure 3:**
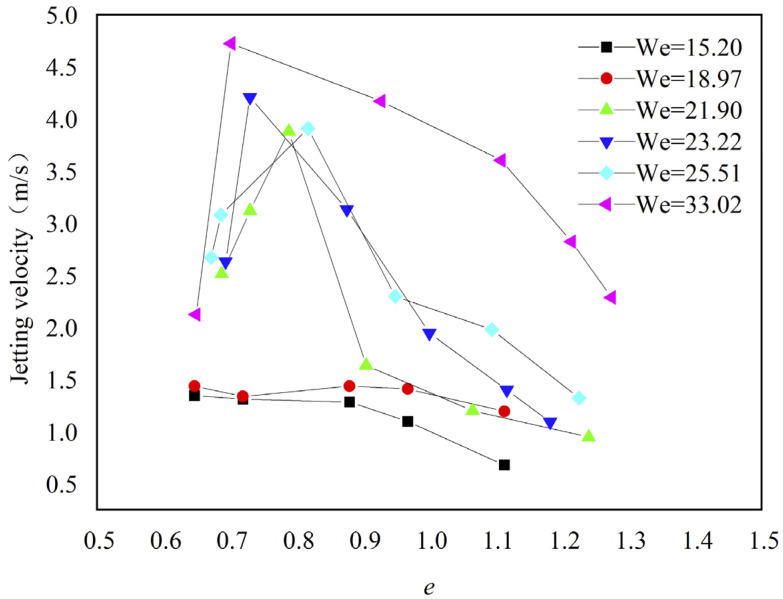
The jetting velocity under different conditions.

To understand the jetting phenomenon, we plotted the jetting velocity in variation with the Weber number We and relative eccentric distance *e* in Figure 3. The jetting velocity was determined using image analysis by which the change in the position of the jetting tip was divided by the time interval between two successive frames of the captured video. The jetting velocity increased as the Weber number increased. The highest jetting velocity reached about 4.6 m/s when We = 33.02. At different values of We, the jetting velocity increased at first and then decreased, and the maximum jetting velocity occurred at *e* = 0.7–0.8 (Figure 3). At We = 15.20 and 18.97, no jetting occurred when *e* was smaller than ~0.60.

**Figure 4:**
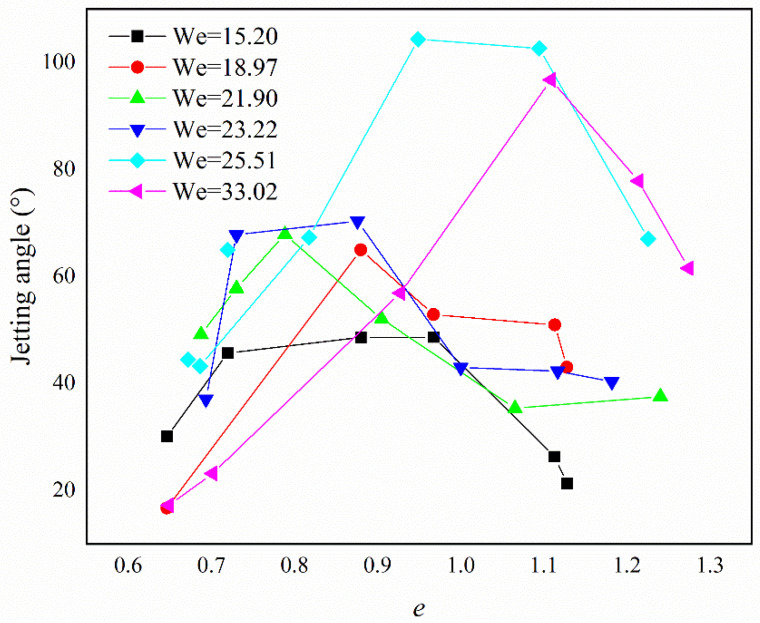
The jetting angle under different conditions.

Figure 4 shows the jetting angle, which increased as the Weber number increased for We < 30. At a fixed value of We, the jetting angle first increased then decreased with the increase in *e*. When the values of We were close to each other, a slight difference in the jetting angle was observed, as shown for the datasets with We = 21.90 and 23.22 in Figure 4.

## 4. Numerical Simulations of Asymmetric Jetting

To unveil the mechanism of asymmetric jetting, we employed Fluent 2020 to simulate the velocity, momentum, and pressure inside the impacting droplet for a deep understanding of the interaction between liquid droplets and solid surfaces.

### 4.1. Model Validation

In validating our numerical model, we used the same water droplet properties as indicated in Section 2, and We = 10.23. The mesh step was set to 0.02 mm, as the grid independence study suggested that the simulation was accurate enough when the mesh step is 0.1 mm in size or finer (Figure S3 in Supplemental Materials).

**Figure 5:**
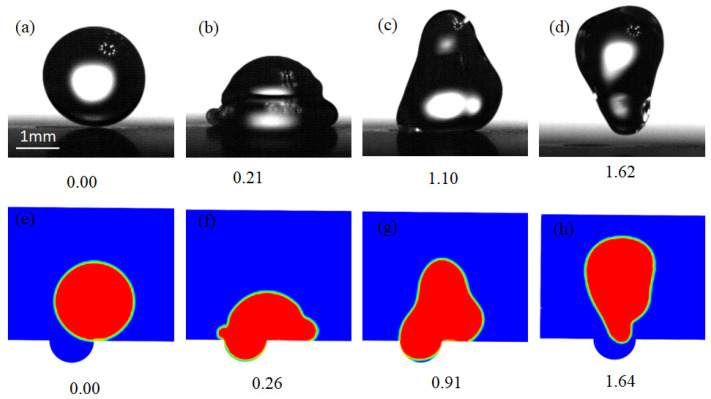
The validation of the simulation model. (**a**–**d**) Experimental results of a droplet impacting a dimpled surface with the normalized time of 0.00, 0.21,1.10 and 1.62, respectively. (**e**–**h**) Simulation of a droplet impacting a dimpled surface with the normalized time of 0.00, 0.26, 0.91 and 1.64, respectively.

Figure 5 contrasts the shape of the droplet from simulations (Figure 5a–d) and experiments (Figure 5e–h), in which the time was normalized by the capillary time $\tau_{cap}$, as defined in Equation (5). Both numerical and experimental results were consistent with each other. For example, at the normalized time of about 0.2 the droplet covered the dimple, and at the normalized time of about 1.6 the droplets took off from the dimpled surface in both experiment and simulation conditions.

(5) $$\tau_{cap}=\sqrt{\rho d_{l}^{3}/\sigma }$$

### 4.2. Simulation Parameters

To simulate a water droplet with a diameter of 2.4 mm that eccentrically impacts the superhydrophobic dimpled surface, the 3D simulation domain was set as 6 mm × 6 mm × 8 mm (length × width × height). The mesh step was 0.02 mm, which was accurate enough from the grid independence study. The unstructured mesh was used in the dimple region, and the structured mesh was used in the air region. The fluid was an incompressible Newtonian fluid. The surface tension of the droplet was 0.072 N/m. The Weber number of the impacting droplet was 21.90. The water contact angle of the solid surface was set to 180° for the removal of any adhesion between the droplet and solid surface. The volume of fluid (VOF) model was used for tracking the two-phase interface. No-slip boundary condition was applied to the solid surface.

### 4.3. Simulation Results

#### 4.3.1. The Flow Field

**Figure 6:**
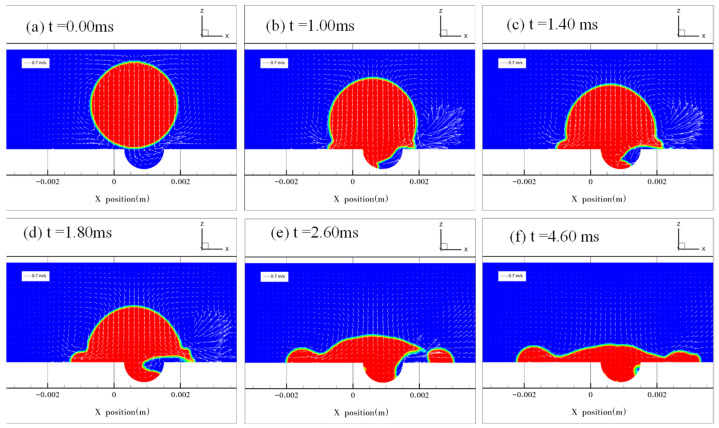
Droplet impacting the dimpled surface at *e* = 0.25. (**a**) The droplet starts to impact the surface when *t* = 0.00 ms. (**b**–**d**) The process of droplet spreading over the dimple, with air trapped inside the dimple. (**b**–**d**) the air is pushed from the center to the right side of the dimple. (**e**) Air evacuated the dimple at t = 2.60 ms. (**f**) The droplet reaches its maximum spreading diameter.

**Figure 7:**
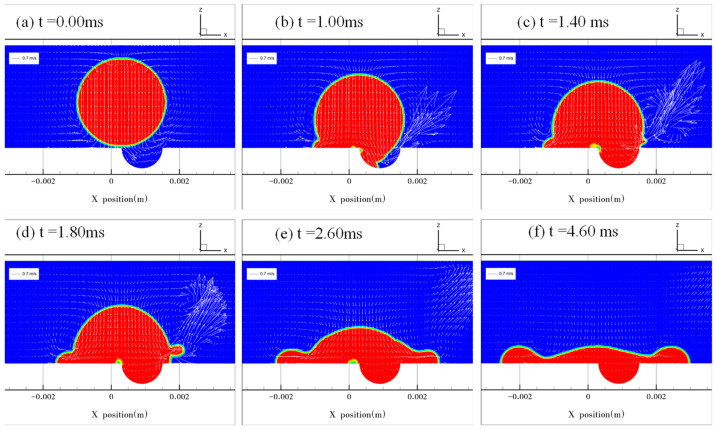
Droplet impacting the dimpled surface at *e* = 0.5. (**a**) The droplet starts to impact the surface when *t* = 0.00 ms. (**b**) The edge of the droplet advances along the dimpled surface. (**c**,**d**) The process of the droplet spreading over the dimple. Jetting is formed on the right side of the droplet. (**e**) The diminishing of jetting due to the surface tension force. (**f**) The droplet reaches its maximum spreading diameter.

As shown in Figure 3, the jetting velocity varied with the change in *e*. As such, we first investigated the dynamic morphological changes of water droplets impacting the dimpled surface with different *e* at We = 21.9. The simulation results are shown in Figure 5, Figure 6 and Figure 7.

Figure 6 shows the results at *e* = 0.25. Figure 6a shows the stage when the droplet made contact with the solid surface at *t* = 0.00 ms. In Figure 6b, the droplet spreads on the surface and covered the dimple with a small volume of air trapped in the dimple. Because the droplet is moving both downward and outward, the droplet spreading pushed the air to the right side of the dimple. If the droplet coaxially impacts the dimple, the underneath air will be locked in the dimple, and it cannot be evacuated until the droplet rebounds because of the symmetric droplet spreading. In contrast, when the droplet impacts the dimple off the center, air could be squeezed out of the dimple at a high speed during the spreading process (Figure 6c–e), leading to a burst of the droplet’s interface (Figure 6e). In Figure 6f, the spreading diameter is maximal. After that, the direction of the flow velocity became reversed, pointing inward and upward, which resulted in bouncing of the droplet. In this case, asymmetric jetting was not observed.

**Figure 8:**
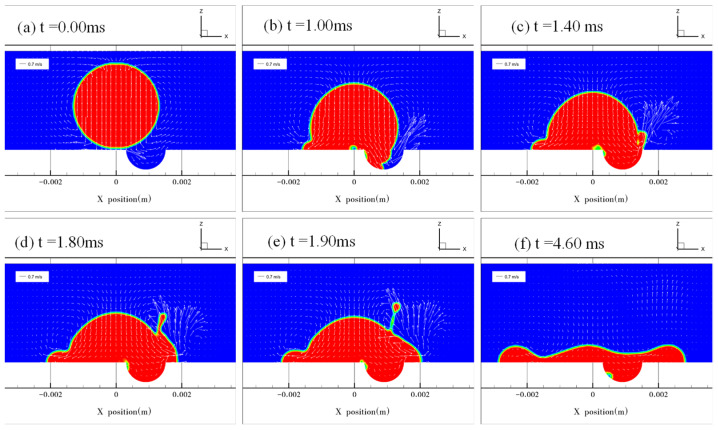
Droplet impacting the dimpled surface at e = 0.75. (**a**) The droplet starts to impact the surface when *t* = 0.00 ms. (**b**) The edge of the droplet advances along the dimpled surface. (**c**) The droplet blankets the dimple. Jetting is formed on the right side of the droplet. (**d**) The jetting position shifts up along the droplet’s surface because of the inertia force. (**e**) The breakup of the jet for the formation of satellite droplets. (**f**) The droplet reaches its maximum spreading diameter.

Figure 7 and Figure 8 show the results at *e* = 0.5 and 0.75, respectively. The droplet contacted the solid surface at *t* = 0.00 ms (Figure 7a and Figure 8a). During droplet spreading on the surface and gradual covering of the dimple, the air in the dimple was evacuated at a high velocity (Figure 7b and Figure 8b,c). When the droplet filled the dimple, the motion of the droplet’s interface at the right edge of the dimple was governed by the superposition of two types of liquid flow in different directions (Figure 7c,d and Figure 8d): one was the liquid flow to the right in the horizontal direction originating from the rightward droplet spreading and another was the vertically upward liquid flow guided by the curvature of the dimple. The presence of the upward liquid flow induced asymmetric jetting at the edge of the dimple. The jetting angle and velocity were thus determined by the magnitude of the two liquid flow velocities. A larger jetting angle and higher jetting velocity are observed in Figure 8 than in Figure 7 because the magnitude of the upward flow velocity is higher in Figure 8, which is consistent with the experimental results in Figure 3.

The simulation unveiled the mechanisms for interface bursting and asymmetric jetting. The bursting was caused by the rapid evacuation of the trapped air inside the dimple (Figure 6) and asymmetric jetting was caused by liquid flow in two directions (Figure 7 and Figure 8). Compared with asymmetric jetting, interface bursting occurred at a smaller value of *e*. As such, a critical value of *e* was required to trigger the occurrence of asymmetric jetting, consistent with the findings in Figure 3.

#### 4.3.2. The Pressure Distribution inside the Droplet

**Figure 9:**
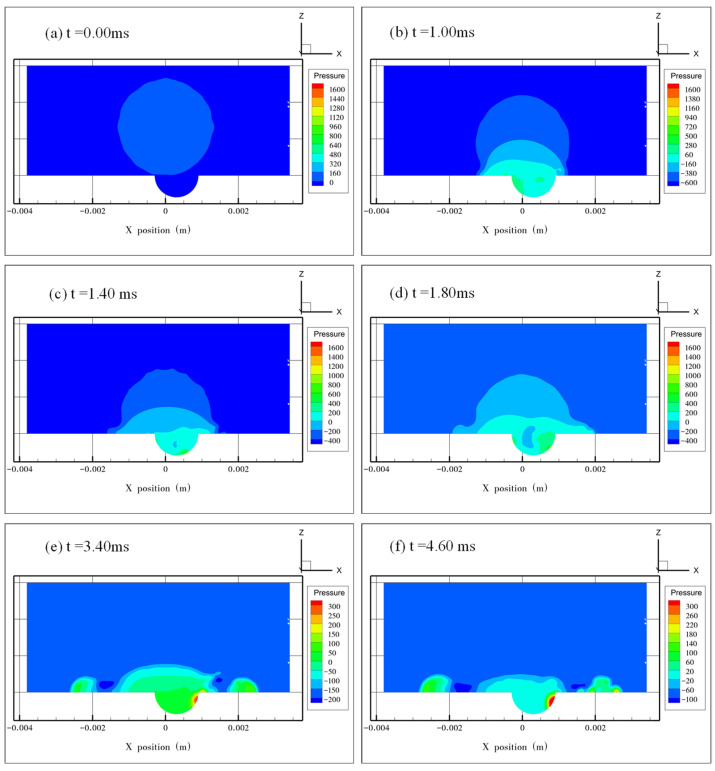
Pressure distribution inside the droplet at *e* = 0.25. (**a**) The initial pressure distribution when the droplet starts to impact the surface at *t* = 0.00 ms. (**b**–**d**) The maximum pressure moves from the left side to the right side of the dimple along with droplet spreading. (**e**) Pressure distribution after the air is evacuated from the dimple. (**f**) Pressure distribution when the droplet spreads to its maximum diameter.

Figure 9 shows the pressure distribution at *e* = 0.25. The initial pressure inside the droplet was about 200 Pa (Figure 9a). In Figure 8b, the maximum pressure (about 800 Pa) occurred at the dimple’s left edge where the droplet first contacted the solid surface. With the spreading of the droplet, the average pressure decreased, and the location of the maximum pressure moved from the left to the right of the dimple. The resulting pressure gradient along the x-direction drove the motion of trapped air from the left to the right inside the dimple (Figure 9b–d). After the droplet’s interface burst, the air was evacuated from the dimple, and the pressure decreased, while the maximum pressure still appeared on the right edge of the dimple, which was about 300 Pa (Figure 9e,f). During this process, there was still some air in the position where the maximum pressure appeared, as shown in Figure 6e,f. Figure 9f also suggests that the maximum pressure inside the droplet occurred at the location where the flow velocity was about 0 m/s, and the minimum pressure occurred at the location where the liquid film was the thinnest.

**Figure 10:**
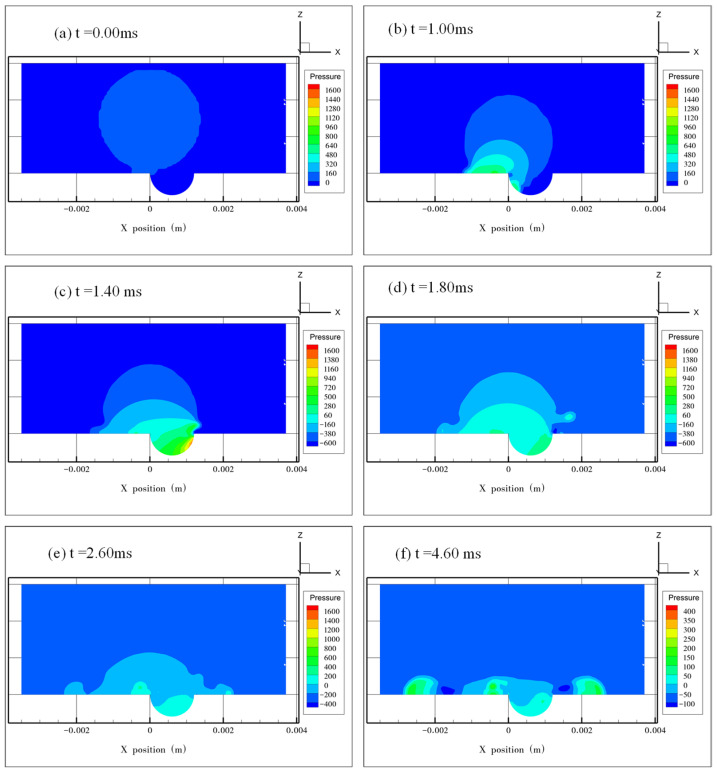
Pressure distribution inside the droplet at e = 0.50. (**a**) The initial pressure distribution when the droplet starts to impact the surface at *t* = 0.00 ms. (**b**–**e**) The maximum pressure moves from the left side to the right side of the dimple along with droplet spreading. In (**d**), the highest pressure (about 60 Pa) occurs in the tip of the jetting finger. (**f**) Pressure distribution when the droplet spreads to its maximum diameter.

**Figure 11:**
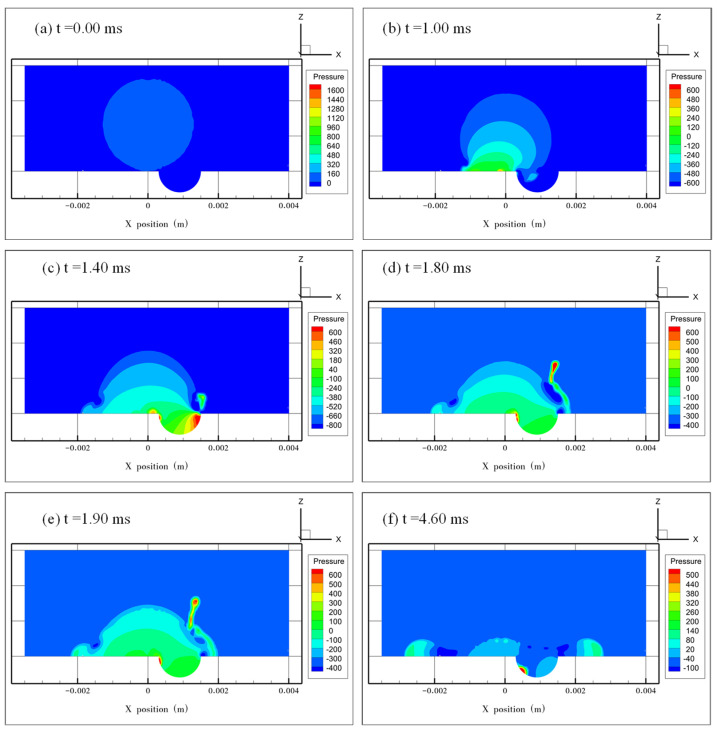
Pressure distribution inside the droplet at *e* = 0.75. (**a**) The initial pressure distribution when the droplet starts to impact the surface at *t* = 0.00 ms. (**b**) The maximum pressure occurs on the liquid–solid interface. (**c**) The maximum pressure shifts from the left side to the right side of the dimple with droplet spreading. (**d**,**e**) The maximum pressure occurs in the tip of the jetting finger during the evolution of the jetting. (**f**) Pressure distribution when the droplet spreads to its maximum diameter.

Figure 10 and Figure 11 show the pressure distribution at *e* = 0.5 and 0.75, respectively. The two cases have a similar tendency in pressure change. At the early stages of droplet spreading, the maximum pressure inside the droplet occurred in the dimple where the air was pushed out. After the jetting was formed, there was no air left in the dimple, and the maximum pressure occurred at the edge of the droplet where the flow velocity was close to 0 m/s. The minimum pressure was located at the position where the liquid film was the thinnest, which is similar to the observation in Figure 9f.

#### 4.3.3. The Momentum Changes in the Droplet

We previously identified the symmetry-breaking of non-specular reflection of impacting droplets and unveiled the momentum difference between droplet impacted on flat and dimpled surfaces [38]. The droplet shape is symmetric at any time instant (see Figure 4), and thus the net momentum is zero in the horizontal direction for the impingement of a droplet on flat surfaces. In sharp contrast, the presence of a dimple affects the internal liquid flow dramatically during droplet impact.

**Figure 12:**
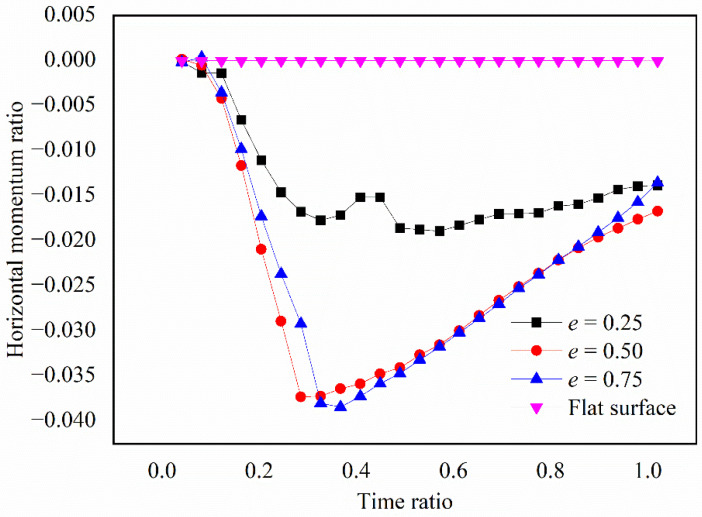
Variations of the horizontal momentum (normalized by the initial momentum at droplet impact) with time (normalized by capillary time).

Figure 12 shows the variations in the horizontal momentum (normalized by the initial momentum at droplet impact) with time (normalized by the capillary time). Here, we defined the positive momentum pointing to the right, and vice versa. On the dimpled surface, the magnitude of the horizontal momentum ratio (MHMR) increased at first and then decreased after the liquid reached the bottom of the dimple. The horizontal momentum ratio varied differently with time between the case of interface bursting and asymmetric jetting. The MHMR of the interface bursting case (at *e* = 0.25) was much smaller than that of the asymmetric jetting case (at *e* = 0.50 and 0.75), which was attributed to the impounded air (at *e* = 0.25) that prevented the accumulation of horizontal momentum during droplet spreading. For asymmetric jetting, the maximum value of the MHMR appeared later when *e* increased, because an increase in *e* indicated a longer distance before the droplet could reach the bottom of the dimple.

## 5. Conclusions

This study identified the asymmetric jetting phenomenon when water droplets impact superhydrophobic surfaces with a macro-sized dimple. The jetting velocity under different conditions was experimentally investigated. Numerical simulations were performed to obtain more details of asymmetric jetting. The following concluding remarks are summarized by combining the results from experimental and numerical studies.

(1) The jetting velocity increases with the increase in the droplet impact velocity. As the eccentric distance increases, the jetting velocity first increases then decreases.
(2) When the eccentric distance is small, interface bursting is induced by the high-speed evacuation of air from the dimple. With the increase in the eccentric distance, asymmetric jetting is triggered by the superposition of liquid flows in the horizontal and vertical directions. The jetting velocity and jetting angle depend on the eccentric distance.
(3) The pressure inside the droplet decreases during droplet spreading. The maximum pressure first occurs at the dimple’s left edge where the droplet makes contact with the solid surface, and then moves from the left edge to the right edge of the dimple. After the spreading droplet spans over the dimple, the maximum pressure occurs at the location where the impounded air stays for interface bursting and at the location where the flow velocity is close to 0 m/s for asymmetric jetting.
(4) When the droplet impacts the flat surface, the net horizontal momentum is zero because of the symmetric distribution of fluid flows, whereas the dimple will break down the symmetry of the horizontal momentum, resulting in a non-zero net value. The maximum magnitude of the horizontal momentum appears at the moment when the liquid droplet reaches the bottom of the dimple and is smaller for interface bursting case than asymmetric jetting.

## Data Availability

The data that support the findings of this study are available from the corresponding author upon reasonable request.

## Supplementary Materials

The following supporting information can be downloaded at: https://www.mdpi.com/article/10.3390/mi13091521/s1, Figure S1: Contact angle measurement; Figure S2: The jetting formation process; Figure S3: Grid independence study.
