# Supplementary material for: Asymmetric Jetting during the Impact of Liquid Drops on Superhydrophobic Concave Surfaces
Source: Micromachines (Basel). 2022 Sep 14;13(9):1521. doi: 10.3390/mi13091521 (PMC9501287; doi:10.3390/mi13091521)
Supplement: Supplementary file 1 [file micromachines-13-01521-s001.zip › micromachines-1866917-supplementary.pdf]

Asymmetric Jetting during the Impact of Liquid Drops on Superhydrophobic Concave Surfaces

Chengmin Chen [1,2,3], Hongjun Zhong [3], Zhe Liu [2], Jianchun Wang [1,2], Jianmei Wang [1,2], Guangxia Liu [1,2], Yan Li [1,2] and Pingan Zhu [4,*]

Contact angle measurement

Figure S1 shows the water contact angle. The value is average of 3 measurements.

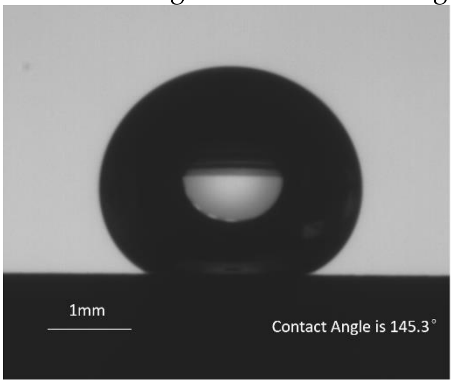

Figure S1. The apparent contact angle of a water droplet.

Jetting formation process captured by camera

Figure S2 is shows the snapshots of the jetting process. At 4k fps, at least four images can be captured for this process, which can be used to analyze the jetting characteristics.

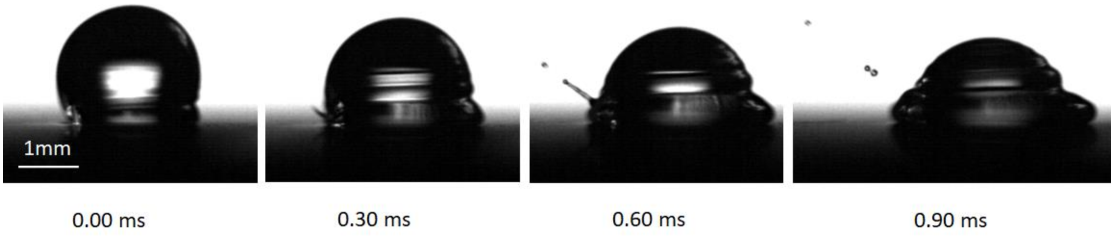

Figure S2. The jetting formation process.

Grid independence study

To figure out the influence of mesh size on the numerical results, four simulations have been run using different mesh sizes, which are 0.20 mm, 0.10 mm, 0.06 mm and 0.02mm respectively. The impacting velocity is 0.45 m/s. Figure S3 shows the fluid velocity in the vertical direction (Z velocity) versus the vertical coordinate (Z position). When the size of the mesh step is smaller than 0.10 mm, the results start to converge. Especially, the discrepancy between the results of 0.06 mm and 0.02 mm is less than 5%. As such, the accuracy of the simulation will be high enough when the mesh step is smaller than 0.10 mm.

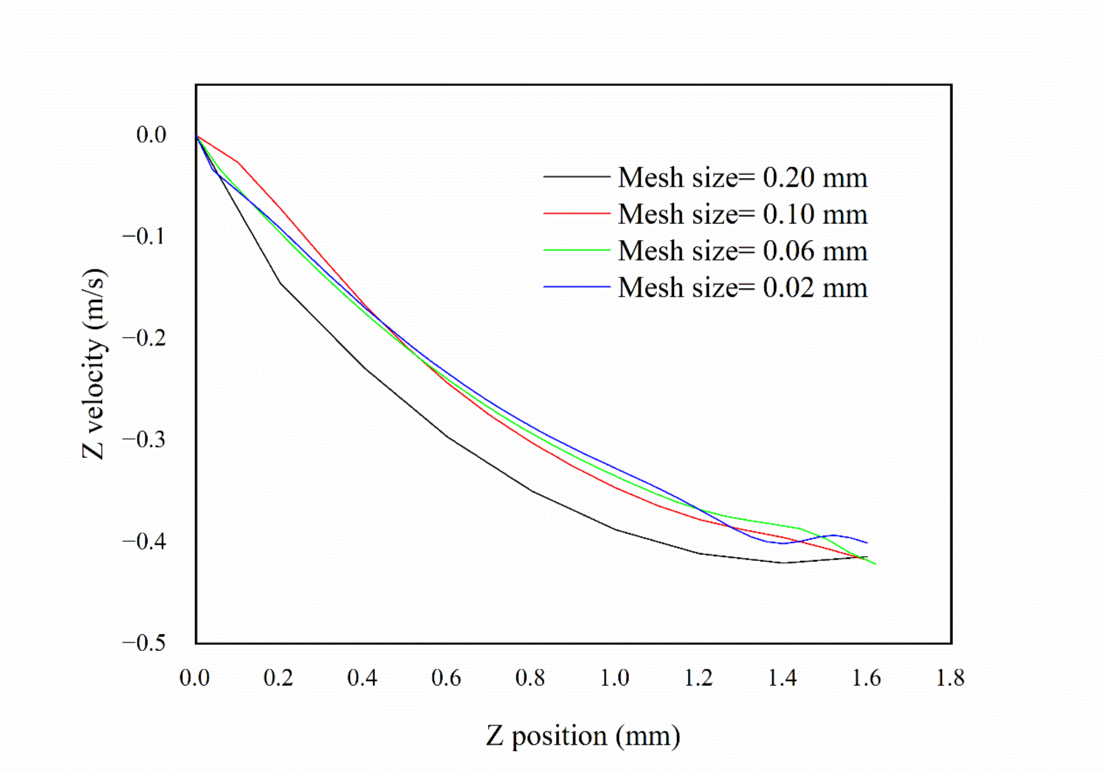

Figure S3. Grid independence study. The Z velocity indicates the fluid velocity in the vertical direction, and the Z position stands for the vertical coordinate.
